# Supplementary material for: Exploring COVID-19 vaccine uptake among healthcare workers in Zimbabwe: A mixed methods study
Source: PLOS Glob Public Health. 2023 Dec 21;3(12):e0002256. doi: 10.1371/journal.pgph.0002256 (PMC10734954; doi:10.1371/journal.pgph.0002256)
Supplement: S1 Table — The table illustrates the results of a multivariable logistic regression sensitivity analysis. The p-value was derived using a likelihood ratio test and the abbreviations: aOR: adjusted odds ratio, 95% CI: 95% confidence interval. (DOCX) [file pgph.0002256.s002.docx]

**Supplementary Table 1: Multivariable logistic regression investigating the association between client characteristics and receiving the vaccine late or never receiving the vaccine (n=1,551)**

| **Variables** | | **aOR** | **95% CI** | **p-value** |
| --- | --- | --- | --- | --- |
| **Sex** | Male | - |  | 0.45 |
|  | Female | 1.08 | 0.89 – 1.31 |  |
| **Age (years)** | < 30 | - |  | < 0.001 |
|  | 30-40 | 0.71 | 0.56 – 0.90 |  |
|  | > 40 | 0.43 | 0.34 – 0.56 |  |
| **Years at current role** | < 1 | 4.08 | 3.19 – 5.22 | < 0.001 |
|  | 1-5 | - |  |  |
|  | 6-10 | 0.96 | 0.76 – 1.22 |  |
|  | > 10 | 1.03 | 0.82 – 1.28 |  |
| **History of SARS-CoV-2 infection** | Yes | 0.83 | 0.52 – 1.32 | 0.43 |
|  | No | - |  |  |
| **Body mass index** | Underweight | 0.99 | 0.60 – 1.63 | 1.00 |
|  | Healthy | - |  |  |
|  | Overweight | 1.00 | 0.81 – 1.22 |  |
|  | Obese | 1.00 | 0.80 – 1.24 |  |
| **Highest level of education** | O-levels | - |  | 0.18 |
|  | A-levels | 0.88 | 0.66 – 1.17 |  |
|  | Diploma | 0.86 | 0.71 – 1.03 |  |
|  | University | 0.78 | 0.60 – 1.01 |  |
| **Administrative authority** | Local | - |  | 0.0004 |
|  | Government | 0.88 | 0.65 – 1.19 |  |
|  | Mission or private | 2.22 | 1.48 – 3.33 |  |
| **Province** | Harare | - |  | < 0.001 |
|  | Bulawayo | 0.82 | 0.63 – 1.07 |  |
|  | Mashonaland East | 2.93 | 1.93 – 4.46 |  |
|  | Mashonaland West | 1.70 | 1.22 – 2.38 |  |
|  | Mashonaland Central | 2.59 | 1.63 – 4.12 |  |
|  | Masvingo | 0.95 | 0.55 – 1.64 |  |
|  | Manicaland | 0.94 | 0.59 – 1.50 |  |
|  | Midlands | 1.20 | 0.75 – 1.93 |  |
|  | Matabeleland South | 0.57 | 0.40 – 0.81 |  |

*p-value was derived using a likelihood ratio test.*

*Abbreviations: aOR: adjusted odds ratio, 95% CI: 95% confidence interval*
