# Supplementary material for: Exploring COVID-19 vaccine uptake among healthcare workers in Zimbabwe: A mixed methods study
Source: PLOS Glob Public Health. 2023 Dec 21;3(12):e0002256. doi: 10.1371/journal.pgph.0002256 (PMC10734954; doi:10.1371/journal.pgph.0002256)
Supplement: S2 Table — The table has three columns showing themes, codes, and relevant supporting quotes that emerged from the qualitative thematic data analysis. (DOCX) [file pgph.0002256.s003.docx]

**Supplementary Table 2: HCWs VACCINE UPTAKE EMERGING THEMES, CODES AND ADDITIONAL QUOTES**

| **Theme** | **Codes** | **Supporting quotes** |
| --- | --- | --- |
| **(Mis)information** | **Government as source of information** | *“Well, the information from the government was that people should vaccinate. That’s how we can be able to suppress the virus”. (Non-clinician, Early Receiver, Harare)* |
|  |  | *“They were encouraging people to get vaccinated… That’s the main message I heard about the vaccine They did not force us…But they are encouraging us to be vaccinated… (Non-clinician, Late Receiver, Harare)* |
|  | **Unclear communication from the government** | *“Yah because when I went there to get the second dose. They realised that there was a big time…Yes, then the other one was like these ones are supposed to start from the first dose. Then the other one was like no, it was decided that we just get them the second dose.” (Clinician, Late Receiver, Matabeleland South)* |
|  |  | *“There was no clear communication. You just heard from colleagues saying there is a new directive. They are now saying it’s this. There wasn’t anything which was like communicated formally.” (Clinician, Early Receiver, Bulawayo)* |
|  | **Social media as the source of information** | *“Personally, I think information was relayed well but was often drawn to social media. And the information conveyed, I think it did their part because we knew everything but we would get confused if you go to social media. Social media is part of our daily life. At one point or the other you will hear something and then you will start debating with what the ministry would have said”. (Clinician, Late Receiver, Matabeleland North).* |
|  |  | *“That social media news that maybe today 100 people got vaccinated and they all died or 3 days after vaccination they died or they reacted badly or something. Very much it had because I delayed getting my vaccine for so long to an extent that I got my vaccination by the time when the government was like, if you don’t get vaccinated you will be kicked out of work or school or something.” (Clinician, Late Receiver, Matabeleland South).* |
|  | **Misinformation from social media and personal interactions with family and friends** | *“…Even though I would want to believe the ministry then by the time I get into social media, it will change the whole idea that I had accumulated through the ministry of health. For example, in South Africa, most of my relatives about 60% they are in South Africa. They had this other vaccine. Most of them they reacted as well, so as a family group we would discuss. You guys this thing is meant to swipe away Africa, let’s not get vaccinated. And then I will also become part of them and just agree that it’s for real.” (Clinician, Late Receiver, Matabeleland North)* |
|  |  | *“So, I was scared, there was this rumour that was saying those people that got vaccinated they just have 3 years to live. In 2023 they will all start dying and be extinct, so I was really scared I don’t want to lie. Those things really pushed me away. Though at some point I would really feel left out because everyone was getting vaccinated.” (Clinician, Late Receiver, Matabeleland North)* |
| **Religion** | **Religious sector as source of (mis)information** | *“They were like neutral and mixed feelings. Like they told people you fear but you should be vaccinated, you should go and get vaccinated. If you feel like you are not ready or whatsoever. Then it’s still your choice to get vaccinated. (Clinician, Early Receiver, Bulawayo)* |
|  |  | *“Well for my church Roman Catholic. They are encouraging people to get vaccinated. They are encouraging people to get vaccinated”. (Non-clinician, Early Receiver, Harare)* |
|  |  | *“Mostly the main reason people are no longer coming it’s because of religion, that is what I perceive to the main problem now”, (Clinician, Early Receiver, Harare)* |
|  | **Religion as a source of myths** | *“About COVID-19 vaccine from the churches they were saying its Satanism. Some saying it’s a way of killing people. Some said if you get vaccinated then in a few years you will get sick and die, so a lot was said. (Clinician, Late Receiver, Mutare)* |
|  |  | *“They didn’t get much into covid issues and getting vaccinated… They did not say much about the vaccine… So on the issue of vaccination, they did not even comment” (Non-clinician, Late Receiver, Westend)* |
|  |  | *“There are those verses in the bible that talk about the beast and 666. That is what was being said that we are being put trackers or something, I don’t know exactly what it was. That’s where a lot of things were said against the covid-19 vaccine.” (Clinician, Late Receiver, Mutare)* |
|  |  | *“We know as Christians we are following also the end time messages that are coming and spiritually people, we are also watching our selves spiritually. So there was a lot of connotations around the vaccines and also to say that it could be also part if the Mark of the Beast. You know and we were so afraid we didn’t know whom to trust, you know.” (Clinician, Early Receiver, Masvingo)* |
| **Perceptions of vaccine efficacy and safety** | **Vaccine origins** | *“Like COVID started from China, the treatment comes from China, how is that possible? Yeah it’s like they are just trying to play with our minds and there are these I don’t know misconceptions or it’s the truth. That 10 years down the line of getting vaccination, you die or have adverse reactions. That China is plotting something that they are not in a hurry to achieve it, so 10 years later they will achieve it. So I don’t know how true is it is.” (Clinician, Late Receiver, Matabeleland South)* |
|  |  | *“Yes, I heard that it was made just to make us infertile so that we do not reproduce as Africans because the Europeans want to rule over Africa something like that, because they wanted our resources. Such things but I didn’t want to become lame to such an extent but those are the things that I read and heard about. And then the other scientists were just wake up and post things like, the Chinese one is fake.” (Clinician, Late receiver, Matabeleland North)* |
|  | **Side effects** | *“Yah after taking the vaccine… headache it was not severe, but it was painful for almost 2 days. Then from others they also had adverse reactions. Others got flu, others had headaches and it validated our issue of saying you actually giving us COVID-19, injecting us COVID-19. Because after getting the vaccine. We had symptoms of COVID-19.” (Clinician, Late Receiver, Matabeleland South)* |
|  |  | *“[T]here is one colleague of ours who reacted to the vaccine. I was really scared because she got into seizures, and she was even transferred. I was so scared even though I really wanted to go and get vaccinated, when I saw her in that state I got scared.” (Clinician, Late Receiver, Matabeleland South)* |
|  |  | *“I don’t understand the vaccine at all, because looking at other countries we would hear some people are getting sick, some are having aftereffects after being vaccinated so people out there” (Clinician, Late Receiver, Harare)* |
|  | **Breakthrough infections** | *“I was saying I can’t get vaccinated because these people that are diagnosed were vaccinated but now, they have been diagnosed they have been infected, so whether vaccinated or not…. We are just the same, that was my argument.” (Clinician, Late Receiver, Matabeleland North)* |
|  |  | *“And there was also this issue of saying even if you are vaccinated you can also be infected, and l also saw many people going to isolation because they had been diagnosed but they got vaccinated. So, I was like what’s the point for getting vaccinated. When you also be infected again. So personally, I was just caught in between, I didn’t know what to believe and what not to believe.” (Clinician, Late Receiver, Matabeleland North)* |
| **SARS-CoV-2 infection and occupational risk** | **High risk work environment** | *“But the greater risk is at work. So, there is very high risk of me contracting COVID as we work with patients every day and you have to touch, and you have to feel sometimes as a nurse.”* (Clinician, Early Receiver, Bulawayo) |
|  |  | *“I feel afraid because the place I work we are around a lot of people”. (Non-clinician, Early receiver, Harare)* |
|  |  | *“It is important [vaccine] because here at the hospital we meet a lot of people. Some come and they are sick of COVID. Some that come for treatment, so you see that you meet a lot of people at the hospital. So the vaccine is important to us” (Non-clinician, Early Receiver, Harare)* |
|  | **Risk outside of work** | *“Some will be presenting signs and symptoms and they want to know about vaccine, some want to know how they can register for ANC, some would be complaining if different issues like stomachache”. (Clinician, Early receiver, Harare)* |
|  | **Inadequate infection and control** | *“so our testing area do not have handwashing facilities, so you have to walk from the tent to come to the laboratory. Yes, you would have removed your regalia but what about other people that you will come across?” (Clinician, Late Receiver, Harare).* |
|  |  | *“And then pull factors that thing that you are working with people that are suffering from COVID-19. And you have nothing to protect yourself, you just feel that you have to go and get vaccinated.” (Clinician, Early Receiver, Bulawayo)* |
|  | **Pre-existing underlying medical chronic conditions** | *“I can easily contract it because of my condition, that I am HIV positive, so we are at risk of contracting a lot of diseases. Because our immune system is weak and is unable to fight strong infections. So that risk makes me afraid that I can contract COVID-19” (Non-clinician, Early Receiver, Harare)* |
| **Employment and access to services** | **Requirement to access services** | *“…, but now those who are coming it’s because they would have heard that they would not get some services, so they are now coming. If you ask that person, they will say, “What can we do, there is nothing we can do, let’s just get vaccinated because I want to go somewhere or because I want this service” (Clinician, Late Receiver, Harare)* |
|  |  | *“My dad was admitted in Harare hospital, but I could not see him because they wanted someone that had 2 doses of vaccination…I couldn’t because they wanted someone that had been vaccinated… So, because of those restrictions... You will just see there is nothing to lose” (Non-clinician, Late Receiver, Harare)* |
|  |  | *“Some of the push factors are that maybe they say that if you are not vaccinated you will not board the ZUPCO buses Or that you won’t be able to enter into the supermarket. Or you won’t be able to go to the bar or you won’t be able to travel from Harare to Bulawayo or Harare to Mutare. Without the vaccination card I feel these are some of the things that will influence people to get vaccinated” (Non-clinician, Early Receiver, Harare)* |
|  | **Mandatory vaccination for work and training** | *“Yah because they were other papers that you were supposed to fill that you got your vaccines, or you are leaving school, or you are losing your job something like that. I don’t know how true it or our bosses were trying to get us vaccinated or something I don’t know.” (Clinician, Late Receiver, Matabeleland South)* |
|  |  | *“A lot of people are being pushed by work because a lot of institutions are saying if you are not vaccinated then we won’t hire you, so that ends up pushing people.” (Non-clinician, Early Receiver, Harare)* |
|  |  | *“What is pushing people mostly it is because nowadays you cannot do anything without being vaccinated, everything you want to do, you have to be vaccinated that is what pushing a lot of people, for you to go to university, to go to work, you need to have a vaccination card, so people realize it is the only way out” (Clinician, Early Receiver, Harare)* |
| **Vaccination confidence and experiences** | **Recommending someone to be vaccinated** | *“Yes, I would recommend as a health care worker, but I tell them they will have to deal with what happens to them later”. (Clinician, Late Receiver, Epworth)* |
|  |  | *“For me the vaccine really helped me so I can recommend its safe (Clinician, Early Receiver, Harare).* |
|  | **Wait and observe other vaccinated individuals (hesitancy)** | *“So, it was an issue of let the people most of the general public get vaccinated and then we hear about the feedback. I didn’t want to be one of the first guys to get vaccinated, I wanted to hear the feedback from the people. From the others. Yes, because we were hearing different type of information that was coming from the public. (Clinician, Late Receiver, Masvingo)* |
|  |  | *“Personally, I did not get vaccinated at the very beginning, I waited to see how others were going to react after 2 weeks. When they came for the second time that is when I was vaccinated”. (Clinician, Early Receiver, Harare).* |
|  | **Vaccination as a positive experiences of others** | *“Yes, they are some people that were saying they are not getting vaccinated at first but now most of us are now vaccinated. I think from the experience that we got. We saw that at first were there was that third wave when it was worse most of the people that would die were the unvaccinated people. So, I think that’s when people started saying that I think we need to get vaccinated.” (Clinician,Early Receiver, Bulawayo)* |
|  |  | *“Our medical superintendent… I think they saw that the attendance of people getting vaccinated was low and they called us all together and they told us the information about the COVID-19 vaccine. Telling us the benefits and highlighting that it is just the same with other vaccines. That people always get injected with and some of our matrons would say I got vaccinated with PMD. So from that time the turnout of people going to get vaccinated was high, that is also the time I went and got vaccinated. But truthfully speaking when the vaccination program started, I didn’t want to. (Clinician, Late Receiver, Mutare).”* |
|  |  | *“Plus, everyone at home had been vaccinated, I was the only one who hadn’t. Yes, my friend is the one that encouraged me. My friend just said bestie go and get vaccinated. Because my friend had been vaccinated as soon as the vaccine came. And my friend would frequently tell me to go but I would ignore. So, my friend then one day sat me down and explained to me, that’s when I decide to go and get vaccinated. My decision was based on the fact that almost everyone was vaccinated, and I was the only one left, so I decided to go and get vaccinated”. (Clinician, Late Receiver, Mashonaland Central)* |
| **Vaccine logistics and access** | **Stock outs** | *“There was a time when the first dose was not available, but the second was always there. Some time back. At the beginning it was available, but there came a time when it was no longer available.”.” (Clinician, Early Receiver, Harare).* |
|  |  | *“Its not always available, sometimes it runs out. Sometimes they say they have run out of first dose; they only have second dose so its vice versa.””. (Clinician, Late Receiver, Harare)* |
|  | **Health facilities as vaccine access points** | *“It helps a lot because there are some people who have a bizarre thinking that if people see them at the clinic, they will think that they are sick.” (Clinician, Early Receiver, Harare).* |
