# Supplementary material for: Exploring COVID-19 vaccine uptake among healthcare workers in Zimbabwe: A mixed methods study
Source: PLOS Glob Public Health. 2023 Dec 21;3(12):e0002256. doi: 10.1371/journal.pgph.0002256 (PMC10734954; doi:10.1371/journal.pgph.0002256)
Supplement: Attachment — Submitted filename: response.pdf [file pgph.0002256.s004.pdf]

I appreciate the opportunity to review your mixed-method study aiming to understand vaccine uptake, perceptions, and attitudes among healthcare workers in Zimbabwe. Below are my comments and suggestions that I believe would enhance the quality and impact of your research.

**Major Points**

Introduction:

- It is imperative for the authors to cite and discuss prior research studies addressing the same research problem. This context will underscore the novelty of your study and help differentiate it from previous attempts.
- Clearly state the aim and objectives of your study at the conclusion of the introduction section to provide a concise overview of your research goals.

Methods:

- In describing your study design as mixed-method, it is crucial to bolster the quantitative component of your manuscript. Authors should employ additional statistical tests (e.g., binary associations, logistic regression models, etc.) to describe and explain the associations between the independent variables and the dependent variable (healthcare workers categorized as receiving the vaccine “early” and those categorized as “late”).
- Authors should provide more information regarding the study population (e.g., what kind of healthcare professionals, students, adults, working facilities, etc.).
- The sampling strategy should be clearly defined and reported in the methods section.
- Authors should clarify how the sample size was determined.
- The authors fail to provide any information in their manuscript that would allow readers to understand how the sample differs from the target population, particularly in terms of key sociodemographic characteristics such as age, gender, and others. This lack of information raises concerns about the representativeness of the sample.
- No information is given regarding a pilot study for testing the survey tools (e.g., questionnaire, interviews, etc.).
- The authors did not provide any information regarding the methods used to collect the data (e.g., questionnaires). Detailed information (e.g., content, type, and number of questions, etc.) on this is crucial for the study’s validity.
- Authors should clarify how they estimated the reliability or internal consistency of the questionnaire used, using, for example, Cronbach’s alpha to measure whether a score is reliable.
- More information should be provided regarding the considerations concerning the literature review (please add the references) and the authors' previous research experience in developing the questionnaire.
- Lines 147-153: Authors need to provide more information and reasoning on how they selected the healthcare professionals that were included in the qualitative study.
- Detailed information should be added about the methods and procedures of the Qualitative Data Analysis (thematic analysis).
- The study should use additional statistical analysis. Specifically, information on how the authors assessed the normality of numeric variables and any relevant assumptions made for the chosen statistical tests or models should be reported to enhance the transparency and reliability of the statistical analysis. Also, information should be added regarding the logistic regression analysis used (e.g., independent and dependent variables, assumptions, etc.)..

Results:

- Detailed findings from the statistical analyses used such as the logistic regression models should be reported in the text of the Results section. The authors should create new tables reporting the results from these analyses, which should be included among the main tables of the manuscript.
- Themes and categories identified from qualitative data analysis should be reported in a corresponding Table.

Discussion:

- Authors should compare their findings not only with studies conducted in Zimbabwe but also with other studies.
- The discussion section should explore the underlying meaning of the research and its potential implications in other areas of study. It would be beneficial for the authors to discuss how their findings contribute to the existing knowledge and highlight the significance of their research within

a broader context. Additionally, they should consider addressing possible improvements or future directions that can further develop the concerns of their research.

- Additional limitations should be added in the limitations section.
- Line 473: It is necessary to include a discussion of the levels of support for a mandatory vaccination policy among HCPs in Zimbabwe and other countries. For example, https://doi.org/10.1016/j.idnow.2021.08.004 , https://doi.org/10.3390/ijerph18136688, https://doi.org./10.3389/fpubh.2022.897526 , https://doi.org/10.3390/vaccines9060580 , https://doi.org/10.3390/vaccines9080889 , https://doi.org/10.3390/hygiene3030021 ) and other articles could be added.

General Points:

- Tables should be reviewed for typos.
- The authors could go through the manuscript once more to correct language errors.
