# Supplementary material for: Exploring COVID-19 vaccine uptake among healthcare workers in Zimbabwe: A mixed methods study
Source: PLOS Glob Public Health. 2023 Dec 21;3(12):e0002256. doi: 10.1371/journal.pgph.0002256 (PMC10734954; doi:10.1371/journal.pgph.0002256)
Supplement: Attachment — Submitted filename: Review Manuscript Number PGPH-D-23-01351.docx [file pgph.0002256.s005.docx]

**PLOS Global Public Health**

**Exploring COVID-19 vaccine uptake among healthcare workers in Zimbabwe:  A mixed methods study**

**Manuscript Number: PGPH-D-23-01351**

**Reviewer Report**

| **Line number (s)** | **Comment** |
| --- | --- |
| Line 73 *Their roll-out has reduced morbidity, severity and deaths [1–3].* | The sentence is a bit short kindly provide more detail. |
| Line 75 and 77 | What is vaccine nationalism? Define it. |
| Line 81 *For instance Zimbabwe, the focus of our research, did not initially sign up…* | Re-read and edit this sentence slightly |
| Line 93 …*high-income and LMICs suggest…* | Write high-income countries instead |
| Line 100 …*priority group with a target set to reach 100% COVID-19 vaccine coverage among this group [18].* | Reach them by which year? |
| Line 102 *…vaccination strategies were successful…* | Which past vaccination strategies? All of them or COVID-19 related? |
| Line 110: *Data was collected as part of a study providing a comprehensive health check to healthcare workers in…* | Would be clearer to say data was collected as part of another study. |
| Line 112: *…interviews were conducted with selected healthcare workers accessing the service between June 2021…* | Accessing what service? |
| Line 126: *The country approved the use of Sinopharm and Sinovac…* | You mean Sinopharm and Sinovac vaccines. |
| Line 124 *Zimbabwe’s COVID-19 vaccination campaign* | Very nicely written section on the history and flow of events |
| Line 137 *…third wave primarily driven by the delta variant which resulted in the highest number of deaths…* | Be more specific. You mean third wave of the COVID-19 pandemic. What is the delta variant? |
| Line 143 Procedures | An overall comment about this section is to sign post where you switch from speaking about a qualitative in-depth interview to a quantitative questionnaire/survey. E.g. on line 151 write “For the **qualitative** in-depth interviews…” to match with line 159 where you state “…as part of the **quantitative** questionnaire…” |
| Line 144 *Following verbal informed consent and prior to accessing the health check…* | What is the health check? Good to detail here at first instance as you go on to mention it further down in this section. |
| Line 146 and 147 *Questions about perceived vaccine safety were based on a WHO survey tool [18].* | Where did you get guidance questions about vaccine uptake? |
| Line 147 and 148 *Written informed consent from selected healthcare workers was obtained for in-depth interviews…* | State how you got informed consent for those who took the questionnaire. |
| Lines 147 to 149 *Written informed consent from selected healthcare workers was obtained for in-depth interviews to better understand healthcare workers reasons for taking or not taking up the COVID-19 vaccine and the challenges they may have faced in the process.* | Best to break up this sentence. First, you mean to say that written informed consent was obtained from all selected healthcare workers. Second, you mean to say that in-depth interviews were used to better understand healthcare workers reasons for taking or not taking up the COVID-19 vaccine and the challenges they may have faced in the process. I recommend you give a citation here on the use of in-depth interviews in research. |
| Line 151 *For the in-depth interviews, healthcare workers were purposively selected…* | Briefly define what it means to purposively select participants and provide citation. |
| Line 153 *A total of 17 in-depth interviews were conducted, after reaching a data saturation point [29].* | Expand briefly on the meaning of data saturation. |
| Line 162 *The interviews were conducted in English, Ndebele, or Shona according…* | Do you mean both the interviews and questionnaires? |
| Line 164 *…all possible probing questions.* | Define what probing questions are and why you needed to use them. |
| Line 173 *…of June 2021, while somebody receiving the first vaccine dose after June 2021 was categorised as “late”.* | Instead of “somebody” write “those” |
| Line 188 *In-depth interviews were audio-recorded, transcribed, and translated. During the interview, research…* | Transcribed and translated by who? |
| Line 191 …software NVivo 12, which was used to perform thematic analysis. Thematic analysis was performed on… | Describe in more detail what thematic analysis is and how you did it. |
| Line 195 *Quantitative findings were triangulated with the themes emerging from the qualitative data throughout the…* | Good starting explanation of mixed methods data analysis but need to detail more. |
| Line 203 *…waived the necessity for a written informed consent to facilitate access to the service.* | How would written informed consent have been a deterrent to access to the service? |
| Lines 438-447 *Beyond the broader patterns of access, availability, and distribution, we identified several socio behavioural influencers that contributed to a comparatively high vaccine uptake.* | Are these socio behavioural influencers also found in published literature? |
| Line 498 *The strengths of this study lie in the use of a mixed-method approach investigating self-reported vaccine…* | Add one more sentence on why mixed -method approach is a strength. |
| Line 503 *…subject to social desirability and/or recall bias (specifically the date of vaccination). Also, healthcare…* | Define social desirability. |
| Line 505 *…may have introduced selection bias as those healthcare workers who took up the health check service…* | Define selection bias. |
| Lines 512-514 *(Mis)information on social media and through religious leaders as well as vaccine-related logistics were also thought to be important.* | Rather than important do you mean could be possible deterrents or barriers to vaccine uptake? |
