# Supplementary material for: Exploring COVID-19 vaccine uptake among healthcare workers in Zimbabwe: A mixed methods study
Source: PLOS Glob Public Health. 2023 Dec 21;3(12):e0002256. doi: 10.1371/journal.pgph.0002256 (PMC10734954; doi:10.1371/journal.pgph.0002256)
Supplement: Attachment — Submitted filename: Manuscript rebuttal letter.docx [file pgph.0002256.s006.docx]

**Mr. Tinotenda Taruvinga**

The Health Research Unit Zimbabwe (THRU ZIM)

Biomedical Research and Training Institute

10, Seagrave Road

Harare, Zimbabwe

E-Mail: [Tinotenda.Taruvinga1@lshtm.ac.uk](mailto:Tinotenda.Taruvinga1@lshtm.ac.uk)

13 October 2023

**Oghenebrume Wariri, MD**

Academic Editor

PLOS Global Public Health

**RE:** **PLOS Global Public Health Decision: Revision required [PGPH-D-23-01351]:** **Exploring COVID-19 vaccine uptake among healthcare workers in Zimbabwe: A mixed methods study.**

Dear Dr. Oghenebrume Wariri,

Thank you very much for considering our manuscript “**Exploring COVID-19 vaccine uptake among healthcare workers in Zimbabwe: A mixed methods study”.** We would like to thank the reviewers for their insightful and detailed comments which we hope that we have satisfactorily addressed in the below point-by-point Response to Reviewers. We believe that addressing these comments has resulted in an improved manuscript and we are grateful to the reviewers for their time. In addition, we have also attached the Inclusivity Questionnaire as requested.

Yours sincerely,

[signature. T. Taruvinga]

Tinotenda Taruvinga, on behalf of the author group

**RESPONSE TO REVIEWERS**

a) **EDITOR’S COMMENTS**

**#Comment 1**

Kindly ensure that you address ALL the issues raised by Reviewers 1 and 2 by providing a point-by-point response on how you addressed the issues in the manuscript. Please pay attention to the issues related to enhancing the quantitative component of the manuscript. Ensure you include supplementary statistical tests (e.g., binary associations, logistic regression models, etc.) to elaborate the connections between the independent variables and the dependent variable, specifically categorizing healthcare workers into "early" and "late" vaccine recipients. Strengthening the quantitative analysis will provide a better understanding of the factors influencing vaccine uptake among healthcare workers, thus contributing to the robustness of the study's findings.

**#Response**

Thank you, for re-emphasizing the need to pay attention to the issues relating to enhancing the quantitative component of the manuscript. We have conducted formal tests to investigate the association between factors such as age and sex and receiving the vaccine late. The analysis has been described in detail in the method section and the results are presented in a table (Table 2); supplementary table 1 and described in the result section. We believe that the enhancement of the quantitative analysis has increased the robustness of the study.

In addition, as we conducted additional analysis on the data, we noticed that we had used data download ending 18 May 2022, instead of data download ending at the end of July 2022. We deeply apologise for this error. We have since done the analysis based on the data download ending July 2022 and the addition of the new data did not change the findings of the study serve for the increased numbers. In that regard we have since made changes on Table 1, Figure 2, and corresponding text to reflect the analysis with additional data. This is well indicated in the tracked version of the revised manuscript from lines 243-271. Just to clarify that table 2 results are based on the data ending July 2022.

b) **REVIWER 1 COMMENTS AND POINT BY POINT RESPONSES**

**#Comment 1**

It is imperative for the authors to cite and discuss prior research studies addressing the same research problem. This context will underscore the novelty of your study and help differentiate it from previous attempts.

**#Response**

Thank you very much for this comment. We have used citations from primary studies to bring forth the context of the study. We believe that this has provided more relevant context to the study.

**#Comment 2**

Clearly state the aim and objectives of your study at the conclusion of the introduction section to provide a concise overview of your research goals.

**#Response**

Thank you for this comment. We have rephrased the last sentence of the introduction. The new sentence now reads, *“In this mixed-methods study, we sought to understand vaccine uptake by describing the ‘early’ and ‘late’ uptake, including qualitatively exploring the perceptions, and attitudes among healthcare workers in Zimbabwe.”* (Line 107-109).

**#Comment 3: Methods**

In describing your study design as mixed method, it is crucial to bolster the quantitative component of your manuscript. Authors should employ additional statistical tests (e.g., binary associations, logistic regression models, etc.) to describe and explain the associations between the independent variables and the dependent variable (healthcare workers categorized as receiving the vaccine “early” and those categorized as “late”).

**#Response**

Thank you for this suggestion. We have conducted a multivariable logistic regression analysis to investigate the associations between socio-demographics, region, and morbidity and early and late vaccine uptake. The result of the analysis are detailed in table 2 and Lines 263-271.

In addition, we have under the data analysis and management (methods) section included information on the additional statistical analysis as suggested (Lines 190-197), “*A multivariable logistic regression was conducted to investigate associations between client characteristics (sex, age, occupation, comorbidities, province of origin, administrative authority, and prior history of SARS-CoV-2 infection) and receiving the vaccine “late”. Variables included in the final model were purposefully selected and analysis was conducted on a complete observation set. P-values were derived using a likelihood ratio test. Results are reported as adjusted odds ratios (aOR) along with their corresponding 95% confidence intervals (95% CI). Those who did not take the vaccine were excluded from the main regression analysis but were however included in the “late” vaccination group in a sensitivity analysis.”*

**#Comment 4**

Authors should provide more information regarding the study population (e.g., what kind of healthcare professionals, students, adults, working facilities, etc.).

**#Response**

Thank you for this comment. We have described the study participants as clinical or non-clinical healthcare workers (Lines 124-125). We provided details about these categories in Lines 202-205, “*Staff roles were categorised into clinical and non-clinical, where clinical staff included, nurses, doctors, laboratory technicians, nurse aides, radiographers, rehabilitation technicians, and pharmacists. Non-clinical staff included administrators, environmental health practitioners, security guards, cleaners, and health information staff*.”

In addition, we described the health facilities healthcare workers were recruited from in Lines 202-208, *“Health facilities were categorised based on their level of care: primary (polyclinics), secondary (district and mission hospitals), tertiary (provincial hospitals) and quaternary (central specialised group of hospitals). These facilities were either owned by local authority, the central government, or faith-based organisations.”* We are unclear what additional detail the reviewer would like to see.

**#Comment 5**

The sampling strategy should be clearly defined and reported in the methods section.

**#Response**

We have added some wording for clarification to Lines 149-152. *“Prior to accessing the health check services, verbal informed consent was obtained. Data on age, sex, professional role, education, clinical history, past SARS-CoV-2 infection and COVID-19 vaccination history, and perceived vaccine safety were obtained from all the healthcare workers accessing the service”.*

Regarding the sampling strategy for the qualitative interviews please refer to Lines 155-160, *“Subsequent to the questionnaire and health check, written informed consent was obtained from a sub-set of healthcare workers purposively selected (i.e. identifying participants sharing particular characteristics not seeking a representative sample but rather a non-probability sample focused on identifying participants fitting the objectives of the study, [30]) for in-depth interviews [29] based on the vaccination status, the time of their first vaccination dose and the place they worked.)”*

**#Comment 6**

Authors should clarify how the sample size was determined.

**#Response**

Thank you for this comment. We did not do a sample size calculation prior to including the vaccine question in the ongoing health care check-up study. All healthcare workers who accessed the health check service during the time the vaccine questionnaire was implemented were included in the study. Assuming vaccine uptake of 90% a sample size of 3086 would have given us a 1.06% precision around the prevalence estimate.

**#Comment 7**

The authors fail to provide any information in their manuscript that would allow readers to understand how the sample differs from the target population, particularly in terms of key sociodemographic characteristics such as age, gender, and others. This lack of information raises concerns about the representativeness of the sample.

**#Response**

We agree that there is likely selection bias since only those healthcare workers who came forward to access the health check were included. These participants may be more health conscious which may have resulted in an overestimation of vaccine coverage. This is mentioned in the discussion under limitations, Line 555-559, which states that, *“Also, healthcare workers were self-selected from those who came forward to access the health check-up service. This may have introduced selection as those healthcare workers who took up the health check service may have been more health conscious and thus more likely to be vaccinated.”*

**#Comment 8**

No information is given regarding a pilot study for testing the survey tools (e.g., questionnaire, interviews, etc.).

**#Response**

Thank you for the comment. We acknowledge that we did not pilot test the vaccine questions. Instead, we adopted the WHO questionnaire. This allowed for comparison with other studies. We indicate this in Lines 152-155 “*The quantitative component involved a questionnaire based on a WHO survey tool [18] which asked about COVID-19 vaccinations history and perceived vaccine safety in addition to the already provided socio demographics and clinical history data.*”

For in-depth interviews the interview topic guide was refined using results emerging from interim analysis. This is explained in Lines 215-229, “*Within an overarching grounded theory approach, [29] we used thematic analysis [30] that ran synchronously with, and subsequent to, the collection of data. Interim analysis of an initial set of transcripts to identify patterns within the data, based on which initial themes and working hypotheses were generated. Hypotheses generated from this interim analysis were fed back into the topic guide and subsequent interviews to refine and, where appropriate, change our working hypotheses. Working iteratively back and forth between analysis and data collection, we worked towards progressively broader and more encompassing themes to explain and theorise findings. Quantitative findings were triangulated with the themes emerging from the qualitative data throughout the analysis process to create meaning of the findings. The overall aim of this bottom-up analytic process was to produce a robust, contextual understanding and interpretation of the vaccine uptake among healthcare workers in Zimbabwe.*”

**#Comment 9**

The authors did not provide any information regarding the methods used to collect the data (e.g., questionnaires). Detailed information

**#Response**

Thank you for this comment. We have added the word questionnaire for clarity in Lines 152-155 to read, “*The quantitative component involved a questionnaire based on a WHO survey tool [18] which asked about COVID-19 vaccinations history and perceived vaccine safety in addition to the already provided socio demographics and clinical history data.”*

For details on the qualitative methods please see information provided in Lines 165-173 –“ *A topic guide was developed prior to the interview and included questions about participants’ vaccination status, challenges of accessing vaccines, concerns about vaccine safety, reasons for being vaccinated or not, and sources of information to guide decision-making. During interviews, participants were given a broader remit to discuss more general concerns and anxieties, as well as why there were these concerns in context, including what specifically concerned them as healthcare workers. While the questions asked as part of the quantitative questionnaire specifically asked about personal reasons for not getting vaccinated (if they had not been vaccinated), the in-depth interview guide gave healthcare workers room to express both positive and negative anxieties they had and those their patients, families and communities may have voiced.”*

**#Comment 10**

Authors should clarify how they estimated the reliability or internal consistency of the questionnaire used, using, for example, Cronbach’s alpha to measure whether a score is reliable.

**#Response**

We used a questionnaire designed by WHO to ensure comparability with other studies. The WHO uses reliability tests such as Cronbach’s alpha when designing and testing a new survey/assessment tool, before deploying the tool for use by countries and partners. The questions we used came from surveys and data collection instruments used around the world, to which reliability and validity tests would have been done.

The questions about vaccine safety and perceptions are as follows:

- [Have you ever received the COVID-19 vaccine?]
- Which vaccine type did you receive?
- How many doses have you received?
- When did you receive the last dose?
- [Is the COVID-19 vaccine readily available at your workplace?]
- [Do you know where to go and receive the vaccine?]
- [What makes it difficult for you to receive the vaccine?]
- [How safe do you think the vaccine is for you?]
- [Would you recommend the vaccine to eligible patients?]
- [Do you think your family and friends would like you to receive the vaccine?]
- [Do you think your family and friends would like you to receive the vaccine?]
- [Do you think your community or religious leaders would want you to receive the vaccine?]
- Why did you not receive the vaccine?

**[ ]** Direct verbatim from the WHO survey questionnaire while the other were adjusted to meet specific study objectives.

While we could use reliability tests such as Cronbach’s alpha by comparing the scores between for example the questions about safety of the vaccine and whether or not a healthcare worker would recommend the vaccine to a patient, we feel this is beyond the scope of the study if not an unnecessary repetition.

**#Comment 11**

More information should be provided regarding the considerations concerning the literature review (please add the references) and the authors' previous research experience in developing the questionnaire.

**#Response**

Thank you for the comment. We did not develop the questionnaire; we adopted an existing WHO questionnaire referenced in Lines 152-155- “*The quantitative component involved a questionnaire based on a WHO survey tool [18] which asked about COVID-19 vaccinations history and perceived vaccine safety in addition to the already provided socio demographics and clinical history data.*

**#Comment 12**

Lines 147-153: Authors need to provide more information and reasoning on how they selected the healthcare professionals that were included in the qualitative study.

**#Response**

Thank you for this comment, more information and reasoning on how healthcare workers were selected for the qualitative study is provided in Lines 156-160 – “*Subsequent to the questionnaire and health check, written informed consent was obtained from a sub-set of healthcare workers purposively selected (i.e. identifying participants sharing particular characteristics not seeking a representative sample but rather a non-probability sample focused on identifying participants fitting the objectives of the study, [30]) for in-depth interviews [29] based on the vaccination status, the time of their first vaccination dose and the place they worked*.”

**#Comment 13**

Detailed information should be added about the methods and procedures of the Qualitative Data Analysis (thematic analysis).

**#Response**

Please refer to Lines 217-225 for clarity we have now included a definition of thematic analysis (underlined in the below paragraph): “*Within an overarching grounded theory approach, [29] we used thematic analysis [30] that ran synchronously with, and subsequent to, the collection of data. Interim analysis of an initial set of transcripts to identify patterns within the data, based on which initial themes and working hypotheses were generated. Hypotheses generated from this interim analysis were fed back into the topic guide and subsequent interviews to refine and, where appropriate, change our working hypotheses. Working iteratively back and forth between analysis and data collection, we worked towards progressively broader and more encompassing themes to explain and theorise findings. Quantitative findings were triangulated with the themes emerging from the qualitative data throughout the analysis process to create meaning of the findings. The overall aim of this bottom-up analytic process was to produce a robust, contextual understanding and interpretation of the vaccine uptake among healthcare workers in Zimbabwe.*”

**#Comment 14**

The study should use additional statistical analysis. Specifically, information on how the authors assessed the normality of numeric variables and any relevant assumptions made for the chosen statistical tests or models should be reported to enhance the transparency and reliability of the statistical analysis. Also, information should be added regarding the logistic regression analysis used (e.g., independent and dependent variables, assumptions, etc.)

**#Response**

We would like to thank the reviewer for this insightful comment. Firstly, variables used in the analysis were plotted to understand their distribution. In Table 1, means with associated standard deviations (SD) were computed for normally distributed continuous variables and medians and interquartile ranges (IQR) were analysed for non-normally distributed continuous variables (Methods: lines 181-183).

We have now conducted a multivariable logistic regression analysis with the outcome being “late” vaccination. Given the very small number of participants who reported not be vaccinated, these were excluded from the analysis. A sensitivity analysis was conducted categorising those who did not receive the vaccine as being vaccinated “late”. The analysis was conducted on the dataset with complete entries for the variables of interest.

In addition, we have under the data analysis and management (methods) section included information on the additional statistical analysis as suggested. In Lines 190-197, it reads, “*A multivariable logistic regression was conducted to investigate associations between client characteristics (sex, age, occupation, comorbidities, province of origin, administrative authority, and prior history of SARS-CoV-2 infection) and receiving the vaccine “late”. Variables included in the final model were purposefully selected and analysis was conducted on a complete observation set. P-values were derived using a likelihood ratio test. Results are reported as adjusted odds ratios (aOR) along with their corresponding 95% confidence intervals (95% CI). Those who did not take the vaccine were excluded from the main regression analysis but were however included in the “late” vaccination group in a sensitivity analysis.”*

**#Comment 15 Results**

Detailed findings from the statistical analyses used such as the logistic regression models should be reported in the text of the Results section. The authors should create new tables reporting the results from these analyses, which should be included among the main tables of the manuscript.

**#Response**

Thank you for this suggestion. We have included a table showing the results of the logistic regression: table 2. We have also described the findings of the table in Lines 263-271 as follows: *“In the multivariable model, age, number of years in current role, administrative authority, and province were associated with receiving the vaccine late. Healthcare workers of older age (aOR: 0.48, 95% CI: 0.37 – 0.62) were less likely to receive the vaccine late. Clients who were working in their current role for less than one year (aOR: 4.01, 95% CI: 3.12 – 5.14) or and those working at a mission or private institution (aOR: 2.32, 95% CI: 1.54 – 3.49), however, were more likely to receive the vaccine late. There was evidence of association between province where the health facility was located and time of vaccine uptake, though it varied by province (Table 2).”*

**#Comment 16**

Themes and categories identified from qualitative data analysis should be reported in a corresponding Table.

**#Response**

Thank you for this suggestion, we have included a table with themes, codes, and additional quotes as identified from the data analysis as a supplementary table, we have inserted a sentence on Lines 292-293 which indicate that there are more quotes. “*Please note we have included themes, codes and additional quotes on appropriate codes and themes in supplementary table 2*”.

**#Comment 17 Discussion**

Authors should compare their findings not only with studies conducted in Zimbabwe but also with other studies.

**#Response**

Thank you for highlighting this. We have compared our findings not only with studies conducted in Zimbabwe but also in other countries such as Nigeria, Somalia, Ethiopia, and Uganda. This is reflected in Lines 484-486,- “*The remarkably high (>97%) prevalence of self-reported COVID-19 vaccine uptake in our study is in stark contrast to other studies from Africa which reported COVID-19 vaccine coverage of 33% and 90% in two settings in Nigeria, respectively, 49% in Somalia, 62% in Ethiopia, 69% in Uganda [31–35]*”.

Please also refer to:

Lines 514-515, “*Studies elsewhere have shown the influence of COVID-19 misinformation on public confidence, leading to vaccine scepticism [38,39]*.”

Lines 518-521, “*While our findings show that misinformation did not hugely affect the vaccine uptake among healthcare workers as in other settings [38,40], these concerns need to be taken seriously in future risk communication and community engagement (RCCE) strategies*.”

**#Comment 18**

The discussion section should explore the underlying meaning of the research and its potential implications in other areas of study. It would be beneficial for the authors to discuss how their findings contribute to the existing knowledge and highlight the significance of their research within 2 a broader context. Additionally, they should consider addressing possible improvements or future directions that can further develop the concerns of their research.

**#Response**

We would like to thank the reviewer for these comments. We feel we have highlighted the significance of this study to the broader context by underlying the importance of this research in Lines 476-477, “*Understanding vaccine uptake and the reasons behind it, as well as remaining concerns that were not adequately addressed, is important for developing new vaccine approaches*.”

We have also indicated possible improvements or future directions that will further the concerns of our research by indicating what needs to be done going forward see Lines 554-559, “*Given their centrality to current and future vaccine policy, their active involvement in the development of strategies is key, especially in addressing legitimate concerns which can be packaged in RCCE activities. Increasing vaccine choices and transparency on adverse effects after immunization through surveillance is likely to address healthcare workers’ legitimate concerns and anxieties. Beyond healthcare workers, misinformation disseminated on social media should be addressed proactively by working with influencers such as religious leaders and public figures*.”

**#Comment 19**

Additional limitations should be added in the limitations section.

**#Response**

We have added more limitations in the section (Lines 578-580) “*Our analysis was more on the strength of the association regarding ‘late’ or ‘early’ COVID-19 vaccine uptake among healthcare workers and thus no causal relationship was explored. “*

.

**#Comment 20**

Line 473: It is necessary to include a discussion of the levels of support for a mandatory vaccination policy among HCPs in Zimbabwe and other countries. For example, https://doi.org/10.1016/j.idnow.2021.08.004, https://doi.org/10.3390/ijerph18136688, https://doi.org./10.3389/fpubh.2022.897526 , https://doi.org/10.3390/vaccines9060580 , https://doi.org/10.3390/vaccines9080889 , https://doi.org/10.3390/hygiene3030021 ) and other articles could be added

**#Response**

Thank you for sharing these links to articles. We have gone through these articles, and we found them very interesting, and we have included some of these citations in the discussion. The studies conducted in Cyprus and Germany very much align with our conclusion despite the very different settings. Mandatory vaccination has several short comings. We suggest alternative approaches to enhance uptake among healthcare workers. Please refer to Lines 528-532 which considers the additional references. “ *COVID-19 vaccine mandatory studies in Cyprus and Germany have shown that the majority of healthcare workers were opposed to the concept of mandatory vaccination [43,44]. Mandatory vaccination most certainly does not solve often quite legitimate concerns people may have about new vaccines and treatment but may exacerbate them [45]. Therefore, mandating vaccination needs to be carefully balanced with other interventions.”*

**#Comment 21: General Points**

Tables should be reviewed for typos.

**#Response**

Noted with thanks. We have reviewed tables for typos, and we have corrected accordingly. In addition, we have included details based on the analysis that involved data download ending July 2021.

**#Comment 22**

The authors could go through the manuscript once more to correct language errors.

**#Response**

This is well noted with thanks. We have also reviewed the manuscript to correct the language errors.

c) **REVIEWER 2 COMMENTS AND RESPONSES POINT-BY-POINT**

**#Comment 1**

Line 73 *Their roll-out has reduced morbidity, severity, and deaths [1–3].*

The sentence is a bit short kindly provide more detail.

**#Response**

Thank you for highlighting this. We have provided more detail to the sentence to read, “*Their roll-out has reduced morbidity, severity and deaths as reported in many vaccine effectiveness studies [1–3].* (Lines 75-76)

**#Comment 2**

Line 75 and 77

What is vaccine nationalism? Define it.

**#Response**

We have rephrased the sentence to include the definition of vaccine nationalism in the manuscript to read, “*However, the hoarding of vaccines by wealthier countries (i.e. ‘vaccine nationalism’) and global unequal vaccine distribution limited the availability of COVID-19 vaccines, especially in low- and middle-income countries (LMICs) [4,5], resulting in slow and often erratic roll out [5]*.” (Lines 76-79).

**#Comment 3**

Line 81 *For instance Zimbabwe, the focus of our research, did not initially sign up…*

Re-read and edit this sentence slightly.

**#Response**

We have slightly edited the sentence to now read, “*For instance, Zimbabwe the focus of our research, did not initially sign up to the COVAX initiative and instead obtained vaccines through bilateral arrangements with China, Russia and India [4,7].* (Line 83-85)

**#Comment 4**

Line 93 …*high-income and LMICs suggest…*

Write high-income countries instead.

**#Response**

Thank you for the suggestion. This has been corrected to read, *“high-income countries”*, as suggested.

**#Comment 5**

Line 100 …*priority group with a target set to reach 100% COVID-19 vaccine coverage among this group [18].*

Reach them by which year?

**#Response**

The Africa CDC target does not have a timeline. We have indicated in brackets that the timeline for the target is not specified, the Lines now read as follows, “*Related to the new recommendations, Africa CDC in their new strategy have maintained healthcare workers as a priority group with a target set to reach 100% (timeline for target unspecified) COVID-19 vaccine coverage among this group [18]*” (Lines 100-103)/

**#Comment 6**

Line 102 *…vaccination strategies were successful…*

Which past vaccination strategies? All of them or COVID-19 related?

**#Response**

We have added COVID-19 to the past vaccination strategy for clarity. *“In view of this ambitious target of vaccinating all healthcare workers, it is crucial to understand whether past COVID*-*19 vaccination strategies were successful and what could be done to address healthcare workers’ perceptions, anxieties, and concerns to reach the ambitious 100% target.”* Now (Lines 103-105).

**#Comment 7**

Line 110: *Data was collected as part of a study providing a comprehensive health check to healthcare workers in…*

Would be clearer to say data was collected as part of another study.

**#Response**

Thank you for this comment, we have adopted the suggestion so the sentence now reads, “*Data was collected as part of broader study providing a comprehensive health check to healthcare workers in Zimbabwe which has been described in detail elsewhere [19]”* (Lines 113-114)

**#Comment 8**

Line 112: *…interviews were conducted with selected healthcare workers accessing the service between June 2021…*

*Accessing what service?*

**#Response**

Healthcare workers accessing the comprehensive health check services. We have revised the sentence, it now reads, “*A quantitative survey and in-depth interviews were conducted with selected healthcare workers accessing the comprehensive health check service between June 2021 and July 2022*.” Now, Lines (114-116).

**#Comment 9**

Line 126: *The country approved the use of Sinopharm and Sinovac…*

You mean Sinopharm and Sinovac vaccines.

**#Response**

We have added “vaccines” for clarity , “*The country approved the use of Sinopharm and Sinovac vaccines before they had received Emergency Authorization Use (EAU) by the WHO [25], followed by Covax, Sputnik V and Johnson and Johnson [26].”* Now Lines (129-131).

**#Comment 10**

Line 124 *Zimbabwe’s COVID-19 vaccination campaign*

Very nicely written section on the history and flow of events

**#Response**

Thank you.

**#Comment 11**

Line 137 *…third wave primarily driven by the delta variant which resulted in the highest number of deaths…*

Be more specific. You mean third wave of the COVID-19 pandemic. What is the delta variant?

**#Response**

Thank you for this comment. We revised the sentence so that it clearly brings out the description by adding... COVID-19 pandemic third wave…, and we have also done the same for the delta variant. The Lines 139-141 now read, “*The highest number of vaccine doses were administered during the third wave of the COVID-19 pandemic in Africa, which was primarily driven by the more contagious SARS-CoV-2 delta variant, which resulted in the highest number of deaths.”*

**#Comment 12**

Line 143 Procedures

An overall comment about this section is to sign post where you switch from speaking about a qualitative in-depth interview to a quantitative questionnaire/survey. E.g. on line 151 write “For the **qualitative** in-depth interviews…” to match with line 159 where you state “…as part of the **quantitative** questionnaire…”

**#Response**

Thank you for these suggestions. We have changed the section as follows, “*Prior to accessing the health check services, verbal informed consent was obtained for the quantitative arm to collect data on age, sex, professional role, education, clinical history, and past SARS-CoV-2 infection from all the healthcare workers accessing the service.* *The quantitative component involved a questionnaire based on a WHO survey tool [18] which asked about COVID-19 vaccinations history and perceived vaccine safety in addition to the already provided socio demographics and clinical history data. Subsequent to the questionnaire and health check, written informed consent was obtained from a sub-set of healthcare workers purposively selected for in-depth interviews [29] based on the vaccination status, the time of their first vaccination dose and the place they worked. The in-depth interviews were administered using a topic guide. This was to better understand healthcare workers reasons for taking or not taking up the COVID-19 vaccine and the challenges they may have faced in the process.* (Lines 149-162).

**#Comment 13**

Line 144 *Following verbal informed consent and prior to accessing the health check…*

*What is the health check? Good to detail here at first instance as you go on to mention it further down in this section.*

**#Response**

Thank you for this comment. We have referred to details of the health check in our opening lines to this methods sections. We have indicated in Lines 113-114 which reads, “*Data was collected as part of a broader study providing a comprehensive health check to healthcare workers in Zimbabwe which has been described in detail elsewhere [19]*.”

**#Comment 14**

Line 146 and 147 Questions about perceived vaccine safety were based on a WHO survey tool [18].

Where did you get guidance questions about vaccine uptake?

**#Response**

Thank you for highlighting this concern. Questions on vaccine uptake were also based on the WHO survey tool. We have revised the sentence to now read as follows, “*The quantitative component involved a questionnaire based on a WHO survey tool [18] which asked about COVID-19 vaccinations history and perceived vaccine safety in addition to the already provided socio demographics and clinical history data.*” (Lines 152-155)

**#Comment 15**

Line 147 and 148 *Written informed consent from selected healthcare workers was obtained for in-depth interviews…*

State how you got informed consent for those who took the questionnaire.

**#Response**

Thank you for your comment, we have now stated how we obtained consent for the questionnaire in Line 149, “*Prior to accessing the health check services, verbal informed consent was obtained.”*

**#Comment 16**

Lines 147 to 149 *Written informed consent from selected healthcare workers was obtained for in-depth interviews to better understand healthcare workers reasons for taking or not taking up the COVID-19 vaccine and the challenges they may have faced in the process.*

Best to break up this sentence. First, you mean to say that written informed consent was obtained from all selected healthcare workers. Second, you mean to say that in-depth interviews were used to better understand healthcare workers reasons for taking or not taking up the COVID-19 vaccine and the challenges they may have faced in the process. I recommend you give a citation here on the use of in-depth interviews in research.

**#Response**

Thank you for the recommendations. We have changed the wording accordingly. The Lines 156-161 now read, *“Subsequent to the questionnaire and health check, written informed consent was obtained from a sub-set of healthcare workers purposively (i.e. identifying participants sharing particular characteristics not seeking a representative sample but rather a non-probability sample focused on identifying participants fitting the objectives of the study, [30]) selected for in-depth interviews [29] based on the vaccination status, the time of their first vaccination dose and the place they worked. The in-depth interviews were administered using a topic guide.”*

We have also included a citation on the use of in-depth interviews as recommended (see above extracted lines).

**#Comment 17**

Line 151 *For the in-depth interviews, healthcare workers were purposively selected…*

Briefly define what it means to purposively select participants and provide citation.

**#Response**

Thank you for this suggestion, we have briefly defined purposive sampling and have added the following lines, “*i.e., identifying participants sharing particular characteristics not seeking a representative sample but rather a non-probability sample focused on identifying participants fitting the objectives of the study, [30].”* Lines 157-159.

**#Comment 18**

Line 153 *A total of 17 in-depth interviews were conducted, after reaching a data saturation point [29].*

Expand briefly on the meaning of data saturation.

**#Response**

Thank you for this comment. We have added a brief explanation of what is data saturation. The Lines now read, “*A total of 17 in-depth interviews were conducted, after reaching a data saturation point [29]* *(that is when we were no longer generating new information to existing categories).”(* Lines 164-165)

**#Comment 19**

Line 162 *The interviews were conducted in English, Ndebele, or Shona according…*

Do you mean both the interviews and questionnaires?

**#Response**

Yes, English, Ndebele or Shona languages was used for both the interviews and questionnaires. We revised the sentence for clarity to read, “*Both the questionnaire and interviews were conducted in English, Ndebele, or Shona according to the participant’s choice.*”

**#Comment 20**

Line 164 *…all possible probing questions.*

Define what probing questions are and why you needed to use them.

**#Response**

Thank you for your comment, we have defined probing questions and the reason for their use in our study. The sentences added read, “*i.e. questions that enable you to follow up on particular areas of interest related to an overarching theme, some of which might not be predictable in advance [29]. This was done to explore in greater detail respondents’ perceptions and feelings about taking up or not taking up the COVID-19 vaccines.”* (Lines 176-179).

**#Comment 21**

Line 173 *…of June 2021, while somebody receiving the first vaccine dose after June 2021 was categorised as “late”.*

Instead of “somebody” write “those”

**#Response**

Thank you for this suggestion, we have revised this accordingly. The Line now reads, “*…of June 2021, while those receiving the first vaccine dose after June 2021 were categorised…”* now Line 190

**#Comment 22**

Line 188 *In-depth interviews were audio-recorded, transcribed, and translated. During the interview, research…*

Transcribed and translated by who?

**#Response**

Transcribed and translated by trained research assistants. We have edited the Line to read, *“In-depth interviews were audio-recorded, transcribed, and translated by trained research assistants.”* Now Line 213

**#Comment 23**

Line 191 …software NVivo 12, which was used to perform thematic analysis. Thematic analysis was performed on…

Describe in more detail what thematic analysis is and how you did it.

**#Response**

We have included the description of thematic analysis and how we conducted both it with the grounded theory approach in Lines 2116-230 as, “*Within an overarching grounded theory approach, [29] we used thematic analysis [30] that ran synchronously with, and subsequent to, the collection of data. Interim analysis of an initial set of transcripts to identify patterns within the data, based on which initial themes and working hypotheses were generated. Hypotheses generated from this interim analysis were fed back into the topic guide and subsequent interviews to refine and, where appropriate, change our working hypotheses. Working iteratively back and forth between analysis and data collection, we worked towards progressively broader and more encompassing themes to explain and theorise findings. Quantitative findings were triangulated with the themes emerging from the qualitative data throughout the analysis process to create meaning of the findings. The overall aim of this bottom-up analytic process was to produce a robust, contextual understanding and interpretation of the vaccine uptake among healthcare workers in Zimbabwe.*”.

**#Comment 24**

Line 195 *Quantitative findings were triangulated with the themes emerging from the qualitative data throughout the…*

Good starting explanation of mixed methods data analysis but need to detail more.

**#Response**

We have included an additional sentence to detail the importance of using mixed methods analysis. The additional sentence reads, “*The overall aim of this bottom-up analytic process was to produce a robust, contextual understanding and interpretation of the vaccine uptake among healthcare workers in Zimbabwe.”* Line 229-230.

**#Comment 25**

Line 203 *…waived the necessity for a written informed consent to facilitate access to the service.*

How would written informed consent have been a deterrent to access to the service?

**#Response**

Thank you for your question. The health check-up service was set up in June 2020 at the time when there were very strict lock-down conditions. This meant that services could only be provided during limited working hours (details about the implementation of the service are described in a manuscript by Marambire et al currently under review at Plos GH) and on an appointment basis to minimise crowding and allow for social distancing. The services were widely advertised and hence anybody who made an appointment and accessed the service was assumed to have the intention to use the service. Clients were free to access all screening and diagnostic tests or just some of them. They received a laminated short information sheet (which was wiped with alcohol after each client) and were asked for verbal consent which was documented on the electronic form. A waver for written informed consent was sought to allow for easy access and efficient throughput. This in turn allowed the service to run efficiently (which was important because of the limited working hours, but also because healthcare workers were under a lot of work pressure during the COVID-19 pandemic), but also made it clear to clients that this was primarily a service and not a research study.

**#Comment 26**

Lines 438-447 *Beyond the broader patterns of access, availability, and distribution, we identified several socio behavioural influencers that contributed to a comparatively high vaccine uptake.*

Are these socio behavioural influencers also found in published literature?

**#Response**

Thank you for this insightful comment. We have included some discussion about the socio behavioural influencers. See Lines 503-505, “*Beyond the broader patterns of access, availability, and distribution, we identified several socio-behavioural influencers that contributed to a comparatively high vaccine uptake as identified by other studies elsewhere [38,39]*.”

**#Comment 27**

Line 498 *The strengths of this study lie in the use of a mixed-method approach investigating self-reported vaccine…*

Add one more sentence on why mixed -method approach is a strength.

**#Response**

Thank you for your comment, we have added one more sentence on why mixed method approach is a strength in Lines 548-551: *“**Our use of both qualitative and quantitative methods allowed us to combine the strengths of deductive and inductive reasoning, while offsetting the limitations of using exclusively one approach. This complementary approach enabled us to produce a robust and reliable, but also nuanced and contextual understanding of vaccine uptake among healthcare workers in Zimbabwe.”*

**#Comment 28**

Line 503 *…subject to social desirability and/or recall bias (specifically the date of vaccination). Also, healthcare…*

Define social desirability.

**#Response**

We have included the definition of social desirability, “*(by which healthcare workers could have responded in a generally positive way which conforms to their profession)*”. (Lines 572-573)

**#Comment 29**

Line 505 *…may have introduced selection bias as those healthcare workers who took up the health check service…*

Define selection bias.

**#Response**

Thank you for the comment, we have since included the definition of selection bias by explaining the nature of the selection bias in lines 575-576, “*This may have introduced selection* *bias as those healthcare workers who took up the health check service may have been more health conscious and thus more likely to be vaccinated.*”

**#Comment 30**

Lines 512-514 *(Mis)information on social media and through religious leaders as well as vaccine-related logistics were also thought to be important.*

Rather than important do you mean could be possible deterrents or barriers to vaccine uptake?

**#Response**

Thank you for this comment. (Mis)information is not necessarily a barrier to vaccine uptake but could act as a positive influence on vaccine uptake, hence the word important which seeks to address both negative and positive influences.
